# Evaluation of the accuracy of Demirjian's method for estimating chronological age from dental age in Shiraz, Iran: Using geometric morphometrics method

**DOI:** 10.1002/cre2.169

**Published:** 2019-03-04

**Authors:** Masood Kermani, Fatemeh Tabatabaei Yazdi, Matin Abed Haghighi

**Affiliations:** ^1^ Faculty of Dentistry Islamic Azad University of Shiraz Shiraz Iran; ^2^ Faculty of Natural Resources and Environment Ferdowsi University of Mashhad Mashhad Iran

**Keywords:** calcification, chronological age, Demirjian's method, dental age, geometric morphometrics method

## Abstract

Knowing the growth and developmental stages of a child or an adolescent allows for a comparison between their developmental and chronological age. The purpose of this study was to evaluate the accuracy of dental age estimation by Demirjian's method and to examine the applicability of Demirjian's method to Iranian children and adolescents. To do so, we investigated the relationship between chronological and dental age in a total of 158 children (81 female and 77 male) from Shiraz. The present study, for the first time, employs geometric morphometrics, a relatively new technique, to offer an accurate quantitative assessment of Demirjian's method. The correlation coefficient between chronological and dental age showed a significant relationship between dental age and chronological age. The average of the chronological age difference between girls and boys at the time of reaching the same stages of dental development was 0.77 years, which meant girls reached each stage of dental development 9 months earlier than their male peers. According to the results of this study, although the use of Demirjian's method in estimating the age of children in the city of Shiraz has acceptable accuracy, more precise studies are recommended to calibrate the method and develop a table adjusted to reflect development in the Iranian population.

## 1 INTRODUCTION

Knowing the developmental stage of a child or adolescent and comparing it to their expected age can aid in the diagnosis of metabolic diseases and endocrine problems, in addition to applications in forensic medicine and determination of syndromes (Koch & Paulsen, 2001; Stewart, Barber, Troutman, & Wei, 1982). In dentistry, the most important role of studying a person's developmental status is the diagnosis and planning of treatment for orthopedic jaw problems. The type and timing of orthodontic treatment and the prediction of its results are based on the prediction of the time of growth spurt in the jaw and rate and direction of future growth.

All growth‐modification treatments such as use of functional devices such as chincaps and head gears, use of extraoral devices, regaining of space in the arches, and decision making on tooth extraction are possible only after information regarding the condition of an individual's development is obtained (Bishara, 2001; Graber, 2000; Mappes, Harris, & Behrents, 1992; McDonald & Avery, 2004).

So far, different methods such as the study of rate of growth, height or weight growth, appearance of secondary sex characteristics, radiographic examination of the skeletal system, and examination of the condition of teeth have been proposed to determine the stage of development (Stewart et al., 1982).

Into the Kraigman classification, dental age (DA) is covered under the biological age category. However, there are two methods for its evaluation:

1. Clinical observation of teeth, which is the simplest and the least accurate method; and
2. Radiographic examination of teeth and tooth buds (Graber, 2000; Stewart et al., 1982).

The findings of both methods are compared with the standard tables for that particular population, and an estimate of the individual's developmental age is obtained. Different studies show that DA closely correlates with chronological age (CA; Jaeger, 1990; Koch & Paulsen, 2001; Stewart et al., 1982). However, the use of dental indices is only useful from birth to early adolescence (Bishara, 2001).

Dental development indices based on the calcification of the crown and root of teeth are preferred to maturity parameters based on growth rate because these indices are useful not only during the limited period of tooth emergence but also throughout the development and growth of teeth. Clinical observations and use of maturity parameters are further compromised by the main causes of teething not being completely known (Demirjian, Goldstein, & Tanner, 1973). The Demirjian's method utilizes radiographic examination to overcome these challenges.

In 1973, Demirjian A. et al. studied seven permanent teeth in the left side of mandible from 2,928 panoramic radiographs of healthy Canadian–French 3–16‐years‐old children and adolescents, which they used to devise a table of indices and a conversion table (Demirjian et al., 1973; Demirjian & Goldstein, 1976; Koshy & Tandon, 1998a; McKenna, James, Taylor, & Townsend, 2002; Tunc & Koyuturk, 2008). Due to the superimposition of images of adjacent tissues in the development and interpretation of the condition of maxillary teeth, the study by Demirjian et al. was based on the development of mandibular teeth. Owing to the symmetrical development of the teeth of an arch, seven left mandibular teeth were selected as the basis for dental evaluation (Demirjian & Goldstein, 1976).

After the development of Demirjian's method, various studies have been performed to determine the accuracy of this method in various populations in different parts of the world, including China, Norway, and India (Davis & Hagg, 1994; Koshy & Tandon, 1998b; Nykanen, Espeland, & Krogstad, 1998). However, these studies were all based on radiologists' diagnoses of the stages of development of teeth as seen in radiographs described in qualitative terms.

The present study employs geometric morphometrics, a relatively new technique, to offer an accurate quantitative assessment of Demirjian's method.

The geometric morphometrics method has so far been used in biological sciences, as well as in specialized dentistry fields such as orthodontics (Jonke & Freudenthaler, 2007). The advantages of the geometric morphometrics method are its ability to separate the two components of shape and size and to visualize deformation (Zelditch, Swiderski, & Sheet, n.d.).

Additionally, the geometric morphometric method permits performing multivariate statistical analyses, such as principal component analysis to determine the principal components of shape differences, as well as canonical variates analysis, to show group difference and investigate the amount of differences between them. Finally, multivariate statistical analyses make it possible to compare the differences between groups quantitatively and to determine the significance level of differences between groups (Zelditch et al., n.d.; Rohlf, 2012; Tabatabaei Yazdi & Alhajeri, 2018).

Several studies have already used Demirjian's method to evaluate DA. For instance, Leurs et al. (2002) examined Dutch children using this method. In their study, 451 Dutch children between 6‐17 years old were evaluated. Their results showed that the difference between CA and the estimated age in Dutch boys was 4% of a year and 6% of a year in girls. Although this study was accurate for Dutch children, Leurs et al. (2002) proposed adjusted tables and curves to convert Demirjian's developmental indicators into DA (Leurs, Wattel, Aartman, Etty, & Prahl‐Andersen, 2005).

A study conducted in Turkey in 2008 by Tunc and Koyuturk concluded that contrary to Hagg and Matson's research, which stated that the Demirjian's method had a higher accuracy and value for younger children, the largest difference was found in the group, which consisted of 5‐6‐year‐old children. This result may be due to instability in the growth of younger children. In the Turkish population, the difference between DA and CA based on Demirjian's method for boys and girls are 1.43–36% of a year and 1.44–5% of a year, respectively, indicating that Demirjian's method does not apply well for the Turkish population and that the separate standards for the Turkish population must be provided (Tunc & Koyuturk, 2008).

In another study conducted by Al Emran in Saudi Arabia in 2008, 490 panoramic radiographs of patients ranging in age from 8.5 to 16 years old were investigated by a radiologist. The study indicated DA to be higher than CA. A comparison between DA of the studied population and the original study published by Demirjian revealed higher numbers (3% and 4% for boys and girls, respectively) for the Saudi population (Al Emran, 2008).

There are other ways to determine age from teeth, which can be used only in forensic medicine due to their destructive nature. In cases where bodies are discovered several years after death, teeth might be the only remaining parts of the body due to their resistance to decomposition. Such cases highlight the importance of accurate methods for determining the age of individuals on the basis of their teeth.

The aim of this study was to determine if the dental development standards provided by Demirjian can be applied to other population groups, including Iranians, specifically to the population of Shiraz, and in particular people aged 5 to 13 (population studied in this study). We also examined whether there is an acceptable relationship between chronological and DA in this population.

## 2 MATERIALS AND METHODS

### 2.1 Samples

This research is a cross‐sectional, descriptive–analytical study. A total of 158 individuals (81 female and 77 male) were recruited into the study from children visiting one of the specialized clinics in Shiraz between May 2015 and August 2016. Because the ideal age for the treatment of orthopedic problems with removable devices is 2 to 3 years before puberty (ages 8–9 for girls and 10–11 for boys), the age range of the subjects was chosen between 5 and 13 years old.

The subjects were all native Iranians. Subjects were all healthy and had no history of severe systemic illness, long‐term drug use, metabolic or endocrine disorders, or hereditary diseases, and presented no history of abnormalities in their jaw and facial area.

According to the study of Lewis (1991) and Sierra (1987), individuals undergoing orthodontic treatment were eligible for admission. Therefore, patients undergoing orthopedic treatment were studied at the same time as the new patients in the radiology department.

Because only panoramic radiographs were required for orthopedic treatment, no additional imaging was performed on patients. Requesting panoramic radiographs is a standard procedure for different treatments; thus, archival images were used without the need for patients' consent.

### 2.2 DA assessment method

**Figure 1:**
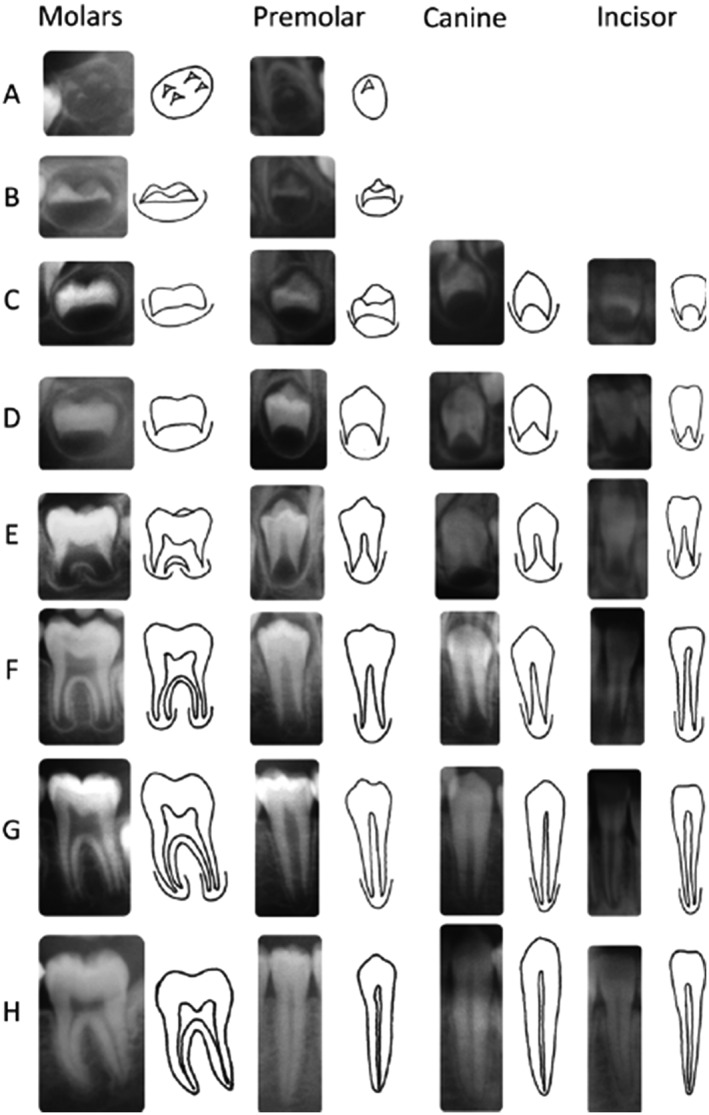
Developmental stages in Demirjian's method(10)

DA was calculated based on the development of teeth on the left side of mandible. First molars and incisors were excluded because their apexes were generally closed in the age interval selected for the study. Therefore, canine, first and second premolars, and second molar teeth were examined in panoramic radiographs and were individually assigned to one of the eight stages defined by Demirjian (A to H; Figure 1).

Orthopantomography (OPG) is one of the most common imaging methods for routine examination in clinical practice. Thus, in the present study, OPG images were used for data gathering (landmarking). OPG scanning was performed in a standard manner using a digital panoramic scanner (Planmeca ProMax 2D), with the minimum exposure time of 16 s, voltage of 66 kV, and current of 9 mA. OPG images were saved in JPG format.

CA was calculated for each patient by subtracting the date of birth from the date of radiography and was recorded in terms of years and month for comparison with the table of ages provided by Demirjian.

**Figure 2:**
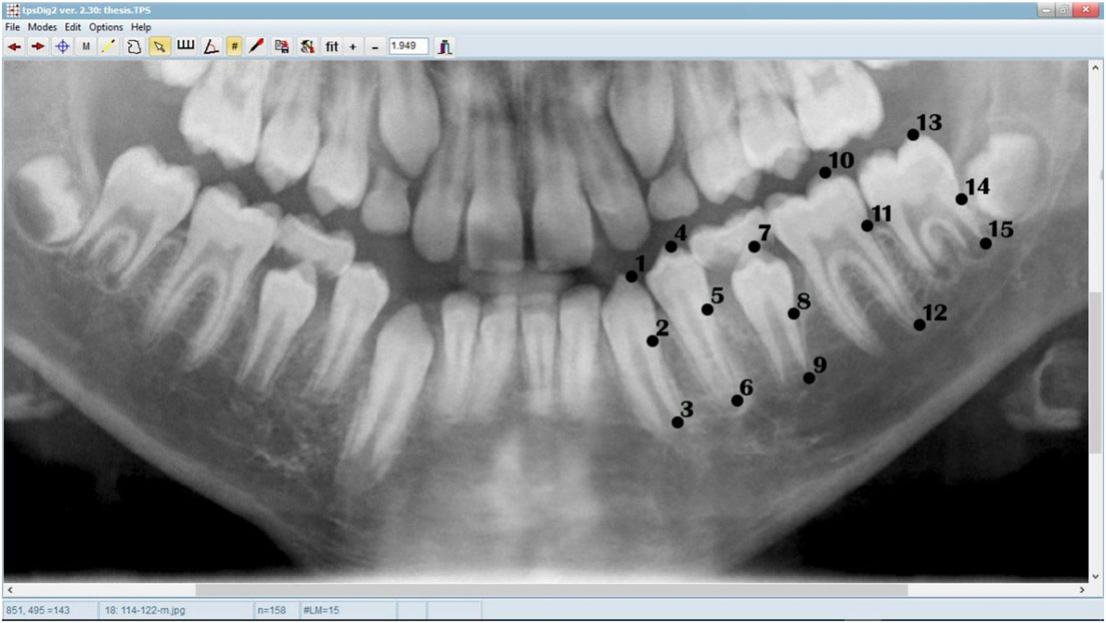
Landmarks in a 12‐years‐old boy

All samples were entered into tpsUtil software (version 1.74) to make a tps file from the images. The tps file was uploaded for landmarking into tpsDig software (version 1.40). The selected landmarks including the tip of cusp, cementoenamel junction, and apex of formed root for each tooth were digitized (Figure 2).

The accuracy of landmarking was confirmed by two expert radiologists at roughly the same time (see the acknowledgement). Using PAST software, the distance between all the landmarks after performing procrust fit (GPA) was calculated. The distances of the required landmarks were imported into Excel 2013. Subsequently, the crown length to root length ratio was calculated.

All of the studied teeth were assigned to one of the developmental stages provided by Demirjian, and respective ages of teeth were calculated using Demirjian's table. DA for each individual was recorded as the mean of the calculated values for each subject's teeth.

### 2.3 Information analysis method

Correlation coefficients and intraclass correlation coefficients were calculated using SPSS (version 22) to evaluate concordance between CA and DA. Descriptive statistical indicators such as percentage and frequency were calculated using SPSS (version 22).

Using SPSS (version 22), the *t* test was performed to compare the mean of CA and mean DA between all age and sex groups.

In order to evaluate the differences between the groups based on Diff and ABS_Diff, analysis of variance (ANOVA) was done using SPSS (version 22).

## 3 RESULTS

The correlation coefficient between CA and DA in this study was 0.856 for boys, 0.891 for girls, and 0.854 for all subjects combined.

The *t* test for the difference between CA and DA shows a significant difference between the mean of difference between DA and CA in both girls and boys. The descriptive indices of the “difference between chronological age and dental age” (Diff) and “absolute difference between chronological age and dental age” (ABS_Diff) show that the mean of Diff in girls is 1.442 (1 year and 5 months), and 0.667 in boys (8 months), indicating DA to be greater than CA for both genders. The mean of the ABS_Diff is 1.479 (1 year and 6 months) in girls and 0.857 (10 months) in boys (Table 1).

**Table 1:** Descriptive indicators of Diff and ABS_Diff variables for each gender

Sex	variable	*N*	Minimum	Maximum	Mean (year)	*SD*
Female	Diff	81	−0.90	3.70	1.442	0.876
	ABS	81	0.10	3.70	1.479	0.811
	Valid N	81				
Male	Diff	71	−1.20	3.30	0.667	0.923
	ABS	71	0.00	3.30	0.857	0.748
	Valid N	71				

Based on the Diff parameter, five groups were formed as follows:

Group 1: The CA 1 to 2 years greater than the DA.
Group 2: The CA zero to 1 year greater than the DA.
Group 3: The DA zero to 1 year greater than the CA.
Group 4: The DA 1 to 2 years greater than the CA.
Group 5: The DA more than 2 years greater than the CA.

**Figure 3:**
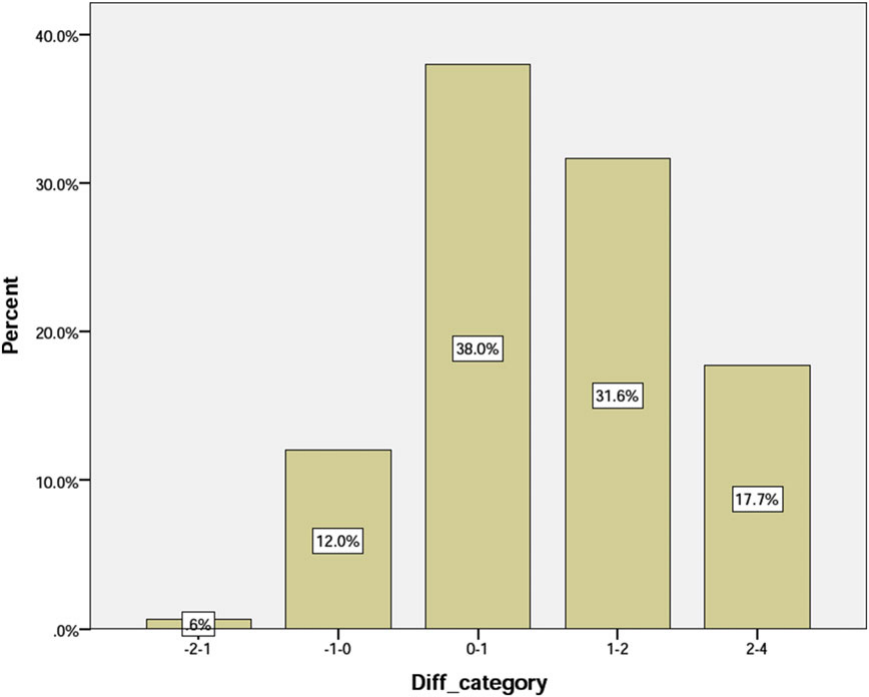
Distribution of the samples based on the Diff category

The analysis of Diff parameter shows that Group 1 has the lowest frequency. In this group, CA is 1 to 2 years greater than DA. The highest frequency is seen in Group 3 where DA is zero to 1 year greater than CA (Table 2). It is also observed that individuals with a greater difference between DA and CA are less frequent (Table 2, Figure 3).

**Table 2:** Abundances based upon Diff category

Group	Diff	Frequency	%	Cumulative %
1	(−2)–(−1)	1	0.6	0.6
2	(−1)–0	19	12.0	12.6
3	0–1	60	38.0	50.6
4	1–2	50	31.6	82.3
5	2<	28	17.7	100.0
	Total	158	100.0	

**Figure 4:**
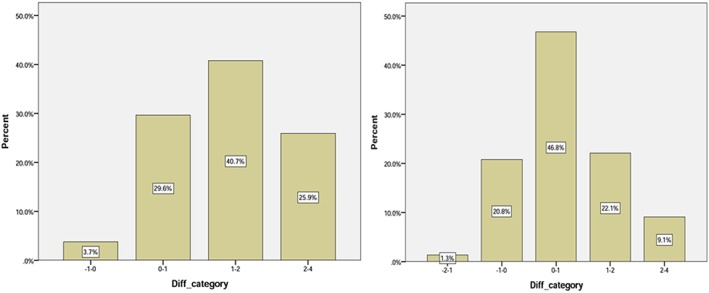
Frequency of the samples in the groups based on the Diff category for girls (left side) and boys (right side)

The comparison of the frequencies between the groups demonstrates that in girls, Group 2 (CA is zero to 1 year greater than DA) is the least frequent, whereas the most frequent group is Group 4 (DA 1 to 2 years greater than CA). Frequencies for all groups are shown in Table 3. It is also noteworthy that groups showing a greater distance between DA and CA are less frequent (Figure 4).

**Table 3:** Gender segregated abundances of the groups, based upon Diff category

Sex	Group	Diff	Frequency	%	Cumulative %
Female	1	(−2)–(−1)	0	0	0
	2	(−1)–0	3	3.7	3.7
	3	0–1	24	29.6	33.3
	4	1–2	33	40.7	74.1
	5	2<	21	25.9	100.0
		Total	81	100	
Male	1	(−2)–(−1)	1	1.3	1.3
	2	(−1)–0	16	20.8	22.1
	3	0–1	36	46.8	68.8
	4	1–2	17	22.1	90.9
	5	2<	7	9.1	100.0
		Total	77	100.0	

Boys in Group 1 (CA 1 to 2 years greater than DA) and Group 3 (DA zero to 1 year greater than CA) are the most and least frequent, respectively. Groups that include cases with a large difference between CA and DA are less populated.

The difference between CA and DA also varies for different age groups. Patients younger than 7 years old showed the smallest difference whereas the largest difference was observed for those between 7 and 9 years old.

A similar trend can be observed for the absolute difference between CA and DA among different age groups (Figure 4). The largest difference can be seen in the 7–9 years age group whereas the smallest value was calculated for subjects older than 11 years old (Table 4).

**Table 4:** Descriptive indices of Diff and ABS_Diff

	Group	Chronological age	*N*	Mean	*SD*	Minimum	Maximum
Diff	1	<7	10	0.57	0.96	−0.90	2.00
	2	7–9	52	1.41	1.08	−1.20	3.70
	3	9–11	43	1.34	0.87	−0.40	3.10
	4	11≤	53	0.58	0.68	−0.90	2.00
		Total	158	1.06	0.97	−1.20	3.70
ABC_Diff	1	<7	10	0.85	0.69	0.10	2.00
	2	7–9	52	1.53	0.90	0.10	3.70
	3	9–11	43	1.36	0.84	0.00	3.10
	4	11≤	53	0.73	0.52	0.00	2.00
		Total	158	1.17	0.83	0.00	3.70

**Figure 5:**
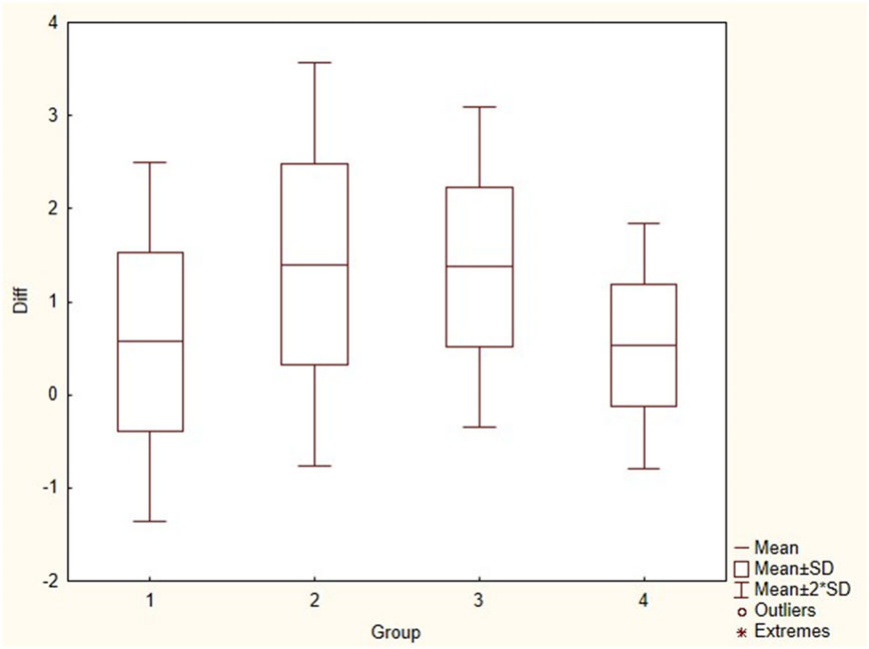
Diff_box plot for different age groups

The results of ANOVA indicated that both Diff and ABS_Diff are significantly different among the four age groups (*p* < 0.001; Figure 5).

According to a pairwise comparison (Table 5), for the Diff variable, age groups of “<7 and 7–9 years”, age groups of “7–9 and 11 ≤ years”, as well as age groups of “9–11 and 11 ≤ years,” showed a significant difference with *p* < 0.05, *p* < 0.001, and *p* < 0.001, respectively. Also, for the ABS_Diff variable, the age groups of “7–9 and 11 ≤ years,” as well as the age groups of “9–11 and 11 ≤ years,” showed a significant difference (*p* < 0.001).

**Table 5:** Multiple comparison of chronological age groups, based on Diff and ABS_Diff

Dependent variable	(I) CA category	(J) CA catgory	Mean difference (I‐J)	*SE*	Sig.
Diff	<7	7–9	−0.84346*	0.31185	0.038
		9–11	−0.77651	0.31707	0.072
		≥11	−0.01679	0.31137	1.000
	7–9	<7	0.84346*	0.31185	0.038
		9–11	0.06695	0.18616	0.984
		≥11	0.82667*	0.17628	0.000
	9–11	<7	0.77651	0.31707	0.072
		7–9	−0.06695	0.18616	0.984
		≥11	0.75972*	0.18536	0.000
	≥11	<7	0.01679	0.31137	1.000
		7–9	−0.82667*	0.17628	0.000
		9–11	−0.75972*	0.18536	0.000
ABS‐Diff	<7	7–9	−0.68654	0.26481	0.051
		9–11	−0.51512	0.26924	0.227
		≥11	0.11981	0.26441	0.969
	7–9	<7	0.68654	0.26481	0.051
		9–11	0.17142	0.15808	0.700
		≥11	0.80635*	0.14969	0.000
	9–11	<7	0.51512	0.26924	0.227
		7–9	−0.17142	0.15808	0.700
		≥11	0.63493*	0.15740	0.000
	≥11	<7	−0.11981	0.26441	0.969
		7–9	−0.80635*	0.14969	0.000
		9–11	−0.63493*	0.15740	0.000

*Note*. CA: chronological age.

## 4 DISCUSSION

According to our findings, the mean difference between CA of girls and boys at the same stages of dental development is 0.77 years, that is, in each stage of dental development, girls are nearly 9 months more developmentally advanced than their male peers. These results are very similar to the results of a study by Heidari (2005), which was carried out in Shiraz. In Heidari's study, the difference in CA between girls and boys was 6 months at each stage of dental development (Heydari, 2005).

According to the results of statistical analyses, in both genders, our subjects reached the end of their developmental pathway earlier than predictions made by Demirjian (1 year and 5 month and 8 month for girls and boys, respectively; 1 year and 1 month for all subjects combined). The same pattern is also observed in other populations, including a study by Sheikhi (2012) in Rasht (Sheikhi & Dakhilalian, 2014).

Based upon the calculated correlation coefficients between CA and DA in this study (0.856 for boys, 0.891 for girls, and 0.854 for all samples), it can be concluded that DA in girls correlates slightly more strongly with CA compared with boys. However, Lamons and Gray (1958), in their investigation in the state of Georgia, found a stronger correlation coefficient between CA and DA (0.96 and 0.95 in boys and girls, respectively; Lamons & Gray, 1958).

In our study, for most subjects, the difference between CA and DA was between zero and 1 year. In both genders, frequency decreases with an increase in Diff, indicating a relative correlation between DA and CA in the majority of the population studied.

The results demonstrate that for the female subjects, the largest difference between CA and DA was observed in the 7–9 age group. This difference could be due to the mean age of appearance of secondary sexual characteristics in Iranian girls being later than the European and African–American girls (Rabbani et al., 2010).

Although, Demirjian's method is the most accepted way to determine status of dental development (Koshy & Tandon, 1998b; Teivens & Mornstad, 2001), for populations other than those used for creating Demirjian's method, population‐specific tables should be generated based on the same features used in the creation of the original table.

## 5 CONCLUSION

The results obtained from this study show that at the same CA, girls show more advanced stages of dental development.

Investigating the relationship between CA and DA in different groups shows that the two ages are positively correlated. In addition, the highest concurrence between dental development and CA can be observed in ages 11 to 13.

According to the results of our study, Demirjian's method for determining DA is acceptable but needs to be modified to match the development of teeth in the Iranian population.
